# pH-Triggered release from surface-modified poly(lactic-*co*-glycolic acid) nanoparticles

**DOI:** 10.3762/bjnano.6.260

**Published:** 2015-12-30

**Authors:** Manuel Häuser, Klaus Langer, Monika Schönhoff

**Affiliations:** 1 Institute of Physical Chemistry, University of Muenster, Corrensstraße 28/30, 48149 Münster, Germany; 2 Institute of Pharmaceutical Technology and Biopharmacy, University of Muenster, Corrensstraße 48, 48149 Münster, Germany

**Keywords:** layer-by-layer self-assembly, pH-triggered release, PLGA nanoparticles, polyelectrolyte multilayers, weak polyelectrolyte

## Abstract

Nanoparticles (NP) of poly(lactic-*co*-glycolic acid) (PLGA) represent a promising biodegradable drug delivery system. We suggest here a two-step release system of PLGA nanoparticles with a pH-tunable polymeric shell, providing an initial pH-triggered step, releasing a membrane-toxic cationic compound. PLGA nanoparticles are coated by polyelectrolytes using the layer-by-layer self-assembly technique, employing poly(acrylic acid) (PAA) as a pH-sensitive component and poly(diallyldimethylammonium chloride) (PDADMAC) as the releasable polycation. The pH during multilayer deposition plays a major role and influences the titration curve of the layer system. The pH-tunability of PAA is intensively investigated with regard to the pH region, in which the particle system becomes uncharged. The isoelectric point can be shifted by employing suitable deposition pH values. The release is investigated by quantitative ^1^H NMR, yielding a pH-dependent release curve. A release of PDADMAC is initiated by a decrease of the pH value. The released amount of polymer, as quantified by ^1^H NMR analysis, clearly depends on the pH value and thus on the state of deprotonation of the pH-sensitive PAA layer. Subsequent incubation of the nanoparticles with high concentrations of sodium chloride shows no further release and thus demonstrates the pH-driven release to be quantitative.

## Introduction

The use of nanoparticles as drug delivery systems has been intensively investigated and important progress has been made within the past decades, establishing reliable methods for particle preparation and characterization. Formation of nanostructures based on different materials, such as metals [1], mineral compounds [2], proteins [3], and a large variety of polymers [4] is widely used in numerous scientific fields. The assembly of biocompatible nanoparticle preparation deserves special attention, if the aim is to apply these nanoparticles as drug delivery systems in vivo. A commonly used polymer fulfilling this criterion is poly(lactic-*co*-glycolic acid) (PLGA), a copolymer consisting of lactic acid and glycolic acid, which has been approved by the authorities to be suitable for pharmaceutical application [5]. Nanoparticles of an appropriate size can be reliably assembled via an emulsion diffusion method, using poly(vinyl alcohol) (PVA) as a stabilizing agent [6]. A part of the stabilizer remains associated with the nanoparticles even after intensive purification procedures because PVA forms an interconnected network with the polymer at the interface [7–8]. PLGA is well-known for its outstanding biocompatibility and its hydrolytic biodegradability which varies in dependence of the relative molar ratio of the monomeric compounds [9]. Subsequent optimization of PLGA-based nanostructures is generally required in order to add specific properties, such as reduced opsonisation [10], a prolonged half-life [11] or improved drug targeting. These optimization procedures are generally performed after particle assembly, since the nanoparticle formation is influenced by many parameters and often limited by minor changes in the experimental setup. However, several further surface modifications are well established these days, such as covalent ligand binding via crosslinking agents [12–13] and different adsorption strategies [14].

The layer-by-layer (LbL) self-assembly technique, introduced by Decher and Hong in the early 1990s [15] has proven to be an outstandingly valuable method for the fabrication of ultrathin polyelectrolyte multilayers (PEM) with well adjustable properties and architecture. Layer build-up is based on alternating adsorption of cationic and anionic compounds, such as polyelectrolytes [15], proteins [16], nucleic acids [17–18], dyes [19], and even colloids [14] onto the surface of charged substrates. The most common and therefore best investigated multilayer build-up involves two oppositely charged polyelectrolytes. Weak polyelectrolytes, such as polyacrylic acid (PAA), with a pH-dependent charge density can be used to add pH-tunable properties to the nanoparticle surface to which they are adsorbed. In case of PAA, a shift of acidity has been reported by several research groups, comparing free polyelectrolyte chains in solution to PAA in multilayers [20], and also the growth behaviour of the layer was shown to depend strongly on the pH value [21]. In the meantime, polyelectrolyte multilayers have already in various cases been applied for pH-driven release, based on weak polyelectrolyte components [22–27].

Nanoparticles can be used as carrier systems for the transport of drugs to cells and tissues. Once getting in contact with cells nanoparticles can be taken up by endocytosis. During the process of endocytosis, nanoparticular drug carriers most often end up in endolysosomes with a reduced internal pH value. In order to provide improved accessibility of the drug to the whole cell, membrane destruction of the endolysosomal bilayer would be beneficial. We present here the idea of a two-step delivery system consisting of core–shell nanoparticles. The outer shell is susceptible to changes of the pH value, such that the release of a membrane-toxic cationic compound is triggered by a reduced pH value in the endolysosomal compartment of the cells. The idea is that this mechanism might in the future trigger a disruption of endosomal membranes and therefore enhance the intracellular distribution of the nanoparticles and the drug that is incorporated in the particle core. In the present work, we employ biodegradable PLGA nanoparticles, which are modified by successive adsorption of four polyelectrolyte layers in total, containing in particular the weak polyanion PAA. We demonstrate that an appropriate pH stimulus induces a controlled release of the polycationic species poly(diallyldimethylammonium chloride) (PDADMAC). The successful pH-triggered release of PDADMAC is concluded from ^1^H NMR spectroscopy. Although ^1^H NMR spectroscopy lacks sensitivity, it has significant advantages with respect to selectivity and robustness compared to current state of the art analytical methods, such as chromatography and titration strategies [28], which are commonly applied to detect anionic polymers [29] or dyes [30]. The obtained release data reveal valuable information about the pH-tunability of PAA in the complexed state within a polyelectrolyte multilayer. The quantification of released PDADMAC is a direct evidence of PEM decomposition and marks their stability limits. In particular, we find the stability limits and the released amount of PDADMA to be dependent on the assembly pH of the multilayers.

## Results and Discussion

### Nanoparticle coating

Successful nanoparticle synthesis can be concluded from measurements of particle size, polydispersity index (PDI), and zeta potential. PLGA nanoparticles were (249 ± 4) nm in diameter, showing a narrow size distribution (PDI 0.04 ± 0.02) and a zeta potential of (−53 ± 1) mV. After adsorption of the initial layer of polyethylenimine (PEI), the particles were washed as described in the Experimental section. The obtained PLGA–PEI nanoparticles showed only a minor increase in particle diameter (266 ± 4) nm and no significant increase in PDI (0.07 ± 0.03), which indicates no aggregation of nanoparticles taking place during adsorption or washing. The zeta potential was inverted to a positive value of (+35 ± 4) mV, which could be attributed to successful adsorption of the cationic PEI layer.

#### pH-Sensitivity of the PAA layer in dependence on the adsorption pH value

PAA, as a weak polyelectrolyte with a charge density depending on pH, can be easily adsorbed onto positively charged substrates, such as PLGA nanoparticles with an adsorbed layer of PEI. In order to investigate the effect of the linear charge density of adsorbing chains on the resulting layer properties, PAA from solutions adjusted to three different pH values, respectively, was adsorbed to PLGA–PEI nanoparticles. Regarding the ionization degree, the titration curve of PAA shows a typical polyelectrolyte behaviour, since instead of a steep slope at the p*K*_a_ value, the degree of ionization smoothly varies over several pH units [20]. The following experiment was carried out adsorbing from PAA solutions adjusted to pH 3 (degree of ionization ≈ 10%), pH 5 (degree of ionization ≈30%), and pH 7 (degree of ionization ≈ 80%), respectively. PAA adsorption was successful in all cases, which can be concluded from the inversion of the zeta potential from positive to negative values (Table 1).

**Table 1:** Zeta potential (ζ) and hydrodynamic radii (*R*_H_) of PAA-coated PLGA-PEI nanoparticles in dependence on pH during PAA adsorption. Nanoparticle radii were obtained in ultrapure water (H_2_O) and 1 mM sodium hydroxide solution (NaOH), respectively. All experiments were performed in triplicate, average values ± standard deviations are shown.

pH (PAA)	ζ/mV (H_2_O)	*R*_H_/nm (H_2_O)	*R*_H_/nm (NaOH)

3	−59 ± 1	130 ± 2	250 ± 2
5	−40 ± 2	126 ± 1	142 ± 2
7	−30 ± 6	126 ± 2	134 ± 5

**Figure 1:**
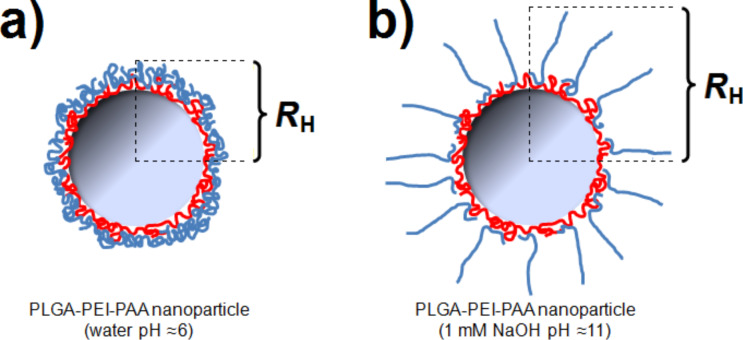
Sketch of terminating layer conformation for PLGA-PEI-PAA nanoparticles prepared at pH 3 in a) ultrapure water (pH approx. 6) and b) in NaOH (pH approx. 11), where PAA is fully charged.

As shown in Table 1, an obvious trend comparing the zeta potential after adsorption to the pH of PAA during adsorption can be extracted. Adsorption of PAA with a low charge density (pH 3) leads to a highly charged nanoparticle system after removal of excess polyelectrolyte. During the washing process using ultrapure water (pH approx. 6), the adsorbed PAA chains become more deprotonated, resulting in a highly charged polyelectrolyte layer on the nanoparticle surface. An opposite effect can be observed considering PAA adsorption at pH 7. Here, the PAA chain is highly charged and therefore adsorbs in a flat, stretched conformation on the particle surface. Incubation with ultrapure water during the washing procedure (pH approx. 6) is not significantly influencing the charge density of PAA on the particle surface, hence leading to considerably lower absolute value of the zeta potential for PAA adsorption at pH 7 as compared to pH 3. This trend can be underlined considering the adsorption of PAA at pH 5, resulting in nanoparticles showing a zeta potential in between the two described cases for PAA adsorption at pH 3 and pH 7, respectively. Regarding nanoparticle radii, no significant differences can be observed by only comparing the determined particle sizes in ultrapure water. In contrast, measurements performed in sodium hydroxide solution (pH 11), show a drastic swelling behaviour for PAA adsorbed at pH 3, but only a slight increase in particle size for PAA adsorbed at pH 5 and pH 7, respectively. This observation can be attributed to different chain conformations of PAA during adsorption, in analogy to the conformation in solution: Due to repulsive forces between the charge-carrying deprotonated carboxylic functions, the PAA chain assumes a stretched conformation under conditions of high pH values and low salt concentrations. On the other hand, a low pH value or the presence of higher amounts of counterions in the polyelectrolyte solution leads to a coiled conformation. Many multilayer studies show that in the former case thin layers are formed, while the latter case yields thicker layers [31]. Several studies have dealt in detail with the influence of salt on layer thickness of polyelectrolyte multilayers [15,32–35] while others described the influence of pH on weak polyelectrolyte layer build-up [36–37]. General concepts of the relevance of the solution conformation have been derived [31]. Concerning charge diluted chains it can be argued that charge compensation, required to compensate the charge density of the terminating layer, determines the surface charge density of the subsequently adsorbing layer. Thus, an adsorbing charge-diluted chain requires a larger amount of mass, yielding thicker films [21,38]. At pH 3, PAA is weakly charged, and therefore adsorbs in a coiled conformation to yield a thick film. In ultrapure water (pH approx. 6), the degree of dissociation in the outermost PAA layer is increasing, causing a layer swelling due to electrostatic self-repulsion. Particularly noteworthy is the massive increase in hydrodynamic radius by more than 100 nm, see Table 1. This implies a significant stretching of single chains after adsorption rather than a swelling of a compact film, as schematically depicted in Figure 1. This behaviour is in agreement with earlier work, where it was shown that particle radii can increase by values on the order of the contour length, when the terminating layer is transferred from high salt to low salt conditions [39].

**Figure 2:**
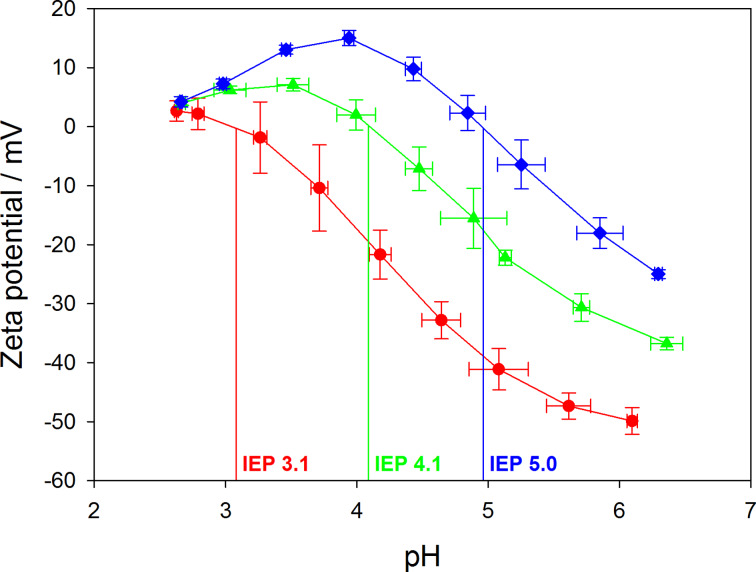
Titration of PLGA–PEI–PAA nanoparticles with HCl (25 mM) for different pH values during assembly of PAA. PAA assembly at pH 7 (blue), PAA assembly at pH 5 (green), PAA assembly at pH 3 (red). Experiments were performed in triplicate, average values ± standard deviations are shown.

When adsorbing PAA at higher pH values (i.e., pH 5 or pH 7), the hydrodynamic radii do not differ much compared to neutral and basic conditions. This can be attributed to the fact that the PAA chains are notably stretched at pH 5 and pH 7 and therefore already adsorb as a flat polymer layer, which remains tightly adsorbed even under basic conditions. Further characterization of the PLGA-PEI-PAA nanoparticles with varying adsorption pH was performed by recording titration curves, where the particle dispersions were titrated with hydrochloric acid (25 mM). By plotting the zeta potential versus pH, the isoelectric point (IEP) can easily be determined. The IEP corresponds to a state of charge neutrality, where ζ is zero. Thus, it is an important parameter characterizing the pH region, in which the particle system is potentially sensitive for pH-triggering. An IEP of unmodified PLGA nanoparticles can be determined in the region of pH 2.2 (data not shown). For coated particles, the IEP depends strongly on the adsorption pH, as shown in Figure 2.

In order to explain the mechanism behind this data, the properties of the PAA layers adsorbed at different pH values have to be taken into account. Considering adsorption at pH 3, PAA adsorbs as a slightly charged chain, forming a thick layer of coiled chains with a high surface coverage of carboxylic functions, as previously explained. The IEP strongly depends on the surface coverage of carboxylic functions. A higher carboxylic surface coverage requires a larger proton concentration in solution to yield neutralisation of the surface layer. On the other hand, at high pH values during adsorption of the PAA layer, a low surface coverage of carboxylic functions is resulting, which can be neutralized already at a higher pH value. The overall probability for complete neutralization of the considered segment on the particle surface at a certain pH value is therefore higher for lower amounts of carboxylic functions in this segment, which can be directly correlated to the pH of PAA during adsorption. In case of the titration curves obtained for PAA adsorbed at pH 5 and pH 7, respectively, a decrease of surface potential is observed after reaching its maximum value (see Figure 2). This can be attributed to surface charge compensation effects induced by the increasing ionic strength, when adding more hydrochloric acid during the titration experiment. In summary, the pH-tunable range of the obtained nanoparticles can be controlled by adjusting the adsorption pH of PAA. It is thus feasible to impose a desired IEP value in the range between pH 3 and pH 5 onto the resulting particle system by choosing an appropriate pH value during assembly.

#### PDADMAC adsorption

**Figure 3:**
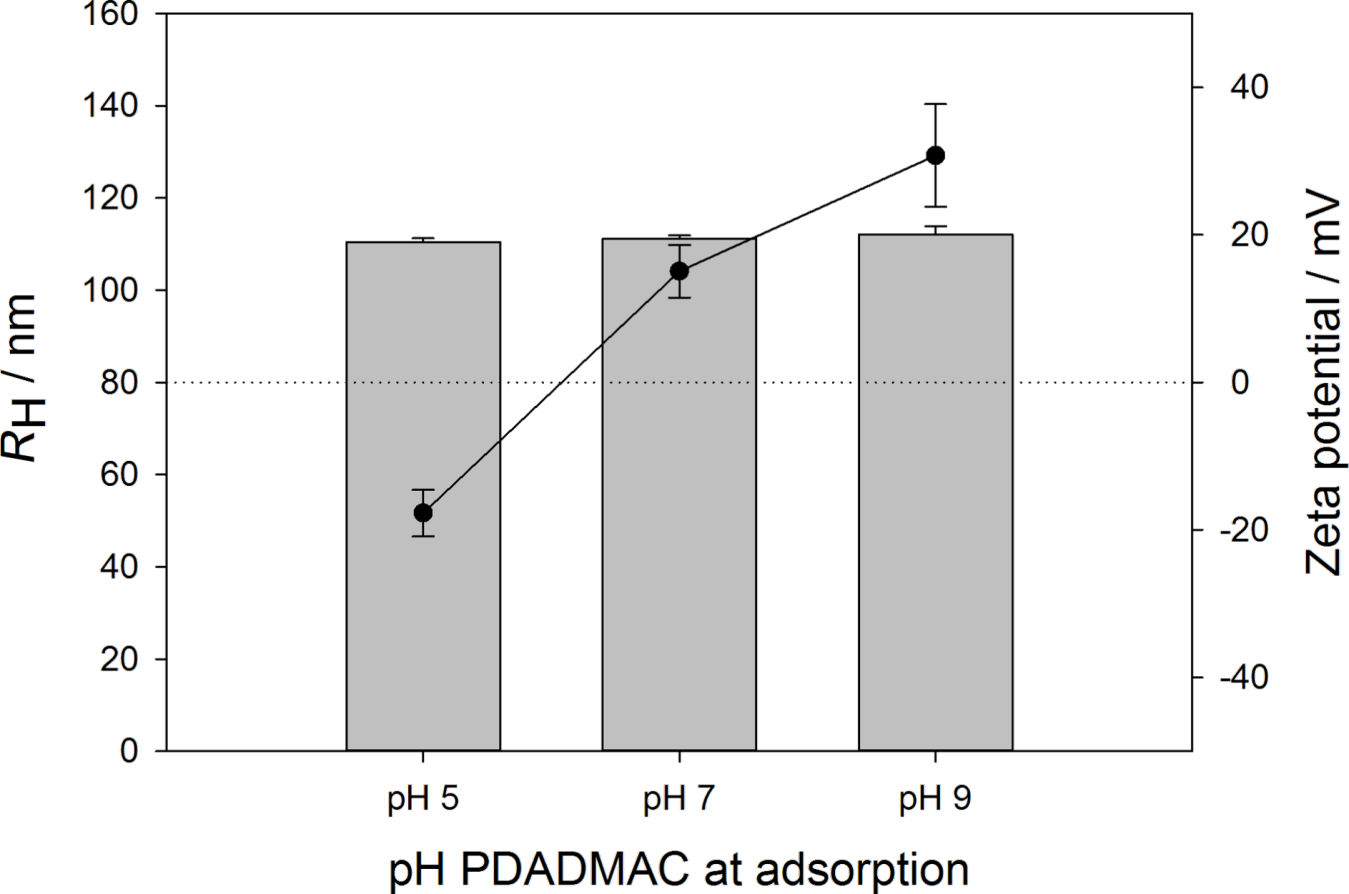
Particle radii (bars) and zeta potential (line plot) of PLGA-PEI-PAA-PDADMAC nanoparticles in dependence of the pH value during PDADMAC adsorption. All experiments were performed in triplicate, average values ± standard deviations are shown.

After intense investigation of the effects concerning PAA modification on the particle system, PDADMAC is subsequently adsorbed in a layer-by-layer fashion. For further layer build-up, the PAA layer has been chosen to be adsorbed at pH 5, forcing the particle system to show an IEP in the region of pH 4. PDADMAC, as a strong polycation, has a charge density that does not depend on the pH value. However, its adsorption might depend on the state of charge of the terminating PAA layer. Therefore, PDADMAC solutions, as well as the PLGA-PEI-PAA nanoparticle dispersions, have been adjusted to pH 5, pH 7, and pH 9, respectively. After adsorption and subsequent washing, nanoparticles are characterized by the hydrodynamic radius and the zeta potential, as determined at pH 6. The dependence of the PDADMAC layer on the pH value during adsorption is shown in Figure 3. All particle samples show a monodisperse size distribution, indicated by a low polydispersity index (PDI ≤ 0.1).

As expected, the nanoparticle size does not depend on the pH value during adsorption of PDADMAC, since the polymer chain conformation of PDADMAC is independent of the pH and therefore always adsorbs as a flat, highly charged layer. In contrast to the particle size, there is a strong influence of the pH value on the zeta potential of PLGA-PEI-PAA-PDADMAC nanoparticles. The pH value of the nanoparticle dispersion before adsorption determines the degree of ionization of PAA on the particle surface and therefore leads to differently pronounced electrostatic attraction to the oppositely charged PDADMAC. The PLGA-PEI-PAA nanoparticle system shows negative zeta potentials at pH 5, pH 7, and pH 9, respectively (data not shown). Adsorption of PDADMAC at pH 5 yields a slightly decreased zeta potential, which still has a negative value of (−18 ± 3) mV, when measured in ultrapure water at pH 6. Apparently, the PAA charge coverage at pH 5 is too low to bind a sufficient amount of PDADMAC, which could neutralize even the charge density of PAA present at pH 6. Only choosing a pH ≥7 during adsorption leads to positive zeta potentials of (+15 ± 4) mV for PDADMAC adsorption at pH 7 and (+31 ± 7) mV for adsorption at pH 9. In summary, PDADMAC adsorption onto PLGA-PEI-PAA nanoparticles significantly depends on the adsorption pH, due to a varying state of deprotonation of the PAA layer on the particle surface, even though PDADMAC provides a charge density of the chain that does not depend on the pH value.

#### Terminating PAA layer adsorption

**Figure 4:**
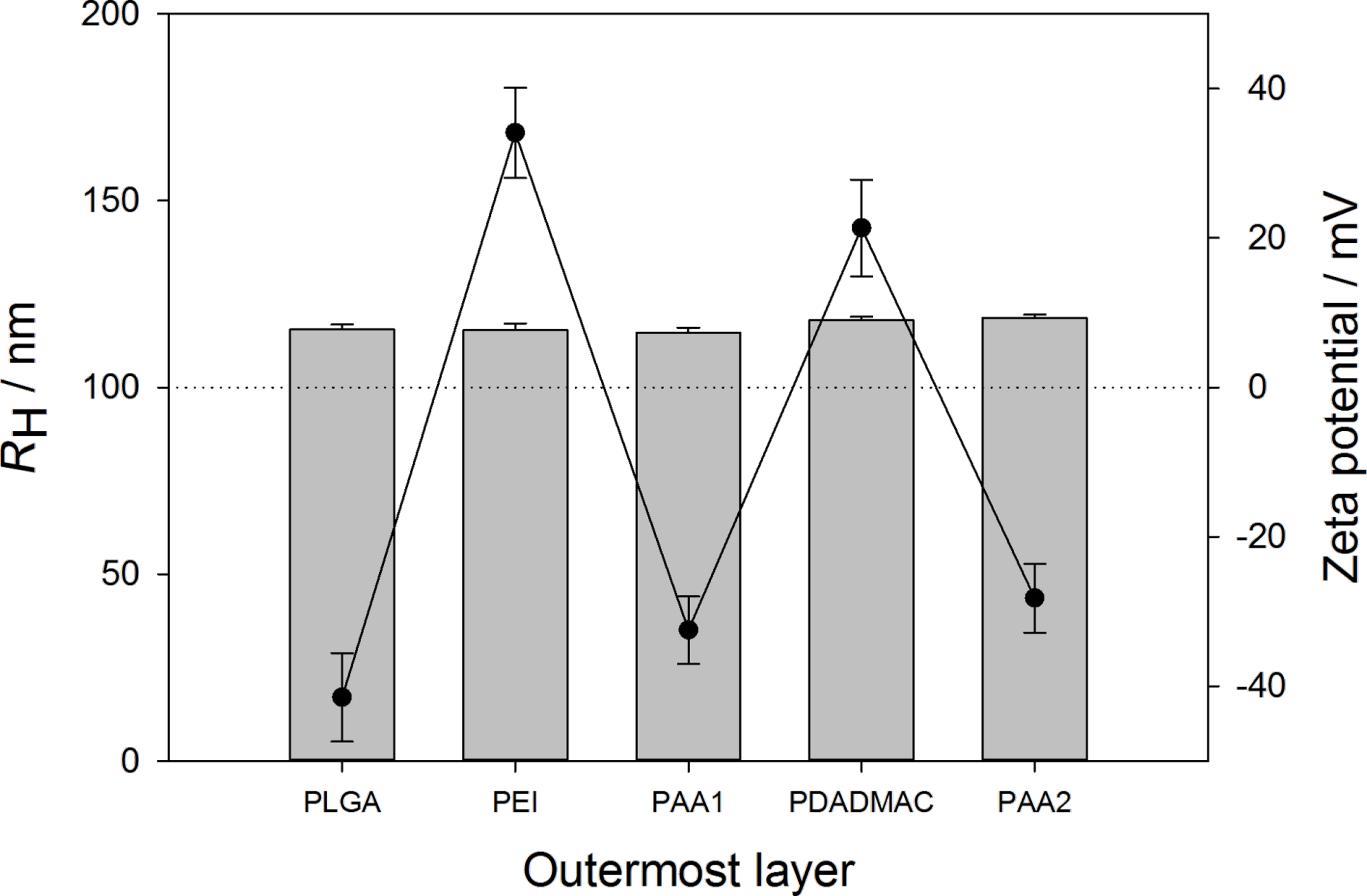
Hydrodynamic radii (bars) and zeta potential (line plot) in dependence of the number of polymer layers for the sequence PLGA-PEI(pH10)-PAA(pH5)-PDADMAC(pH9)-PAA(pH5). All experiments were performed in triplicate, average values ± standard deviations are shown.

The third polymer layer of PDADMAC is followed by another layer of PAA, terminating the particle preparation. Figure 4 shows the resulting particle sizes and zeta potentials after each step of the nanoparticle modification, which underlines the successful preparation, demonstrated by a constant size and an inversion of the zeta potential after each adsorption step.

#### Release of PDADMAC

**Figure 5:**
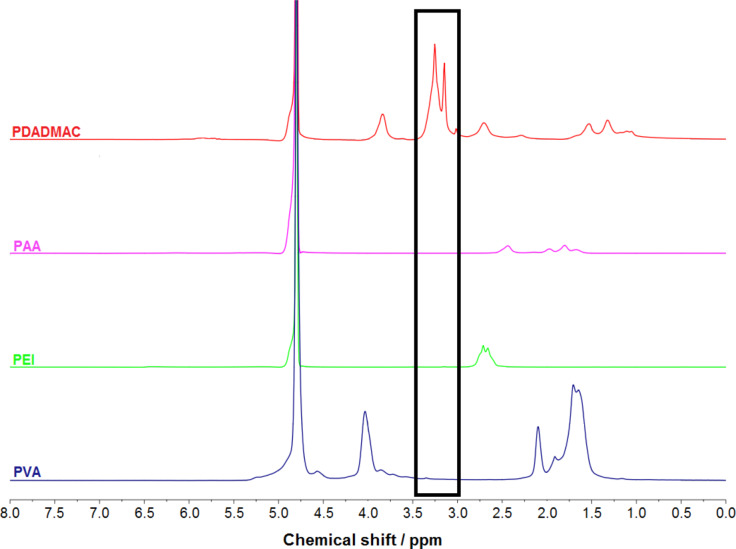
^1^H NMR spectra of solutions (50 mM monomer conc.) of PDADMAC, PAA, PEI, and PVA (from top to bottom). Quantification of PDADMAC in particle dispersion is based on the absence of signals between 2.9 and 3.4 ppm for any other polymer than PDADMAC.

To probe pH-tunability after adsorption of the second PAA layer, a titration experiment using HCl (25 mM) was carried out, as described in the Experimental section. PDADMAC desorption was monitored by quantitative ^1^H NMR spectroscopy, using an external standard of resorcinol in D_2_O as a reference substance. In Figure 5, ^1^H NMR spectra of all polymeric components are shown, except for PLGA, which is insoluble in D_2_O. Recorded spectra are stacked as a guide to the eye, while each polymer spectrum is normalized to the signal of residual water protons (HDO), calibrated by a chemical shift of 4.8 ppm.

Typical broad polymer resonances are observed, where two characteristic signals for PDADMAC occur at chemical shifts of 3.15 and 3.25 ppm in a region where all other polymers show now signal, which makes a quantification of PDADMAC possible using ^1^H spectra. Quantification of PEI or PAA is difficult due to the comparatively lower signal intensities and some spectral overlap. The reference spectrum of resorcinol (data not shown) shows only chemical shifts higher than 4.8 ppm, which do not interfere with any polymer signal. For quantification, the integral of the resorcinol signal at a chemical shift of 7.1 ppm is used and related to the integrals of the PDADMAC signals at 3.15 and 3.25 ppm, respectively.

**Figure 6:**
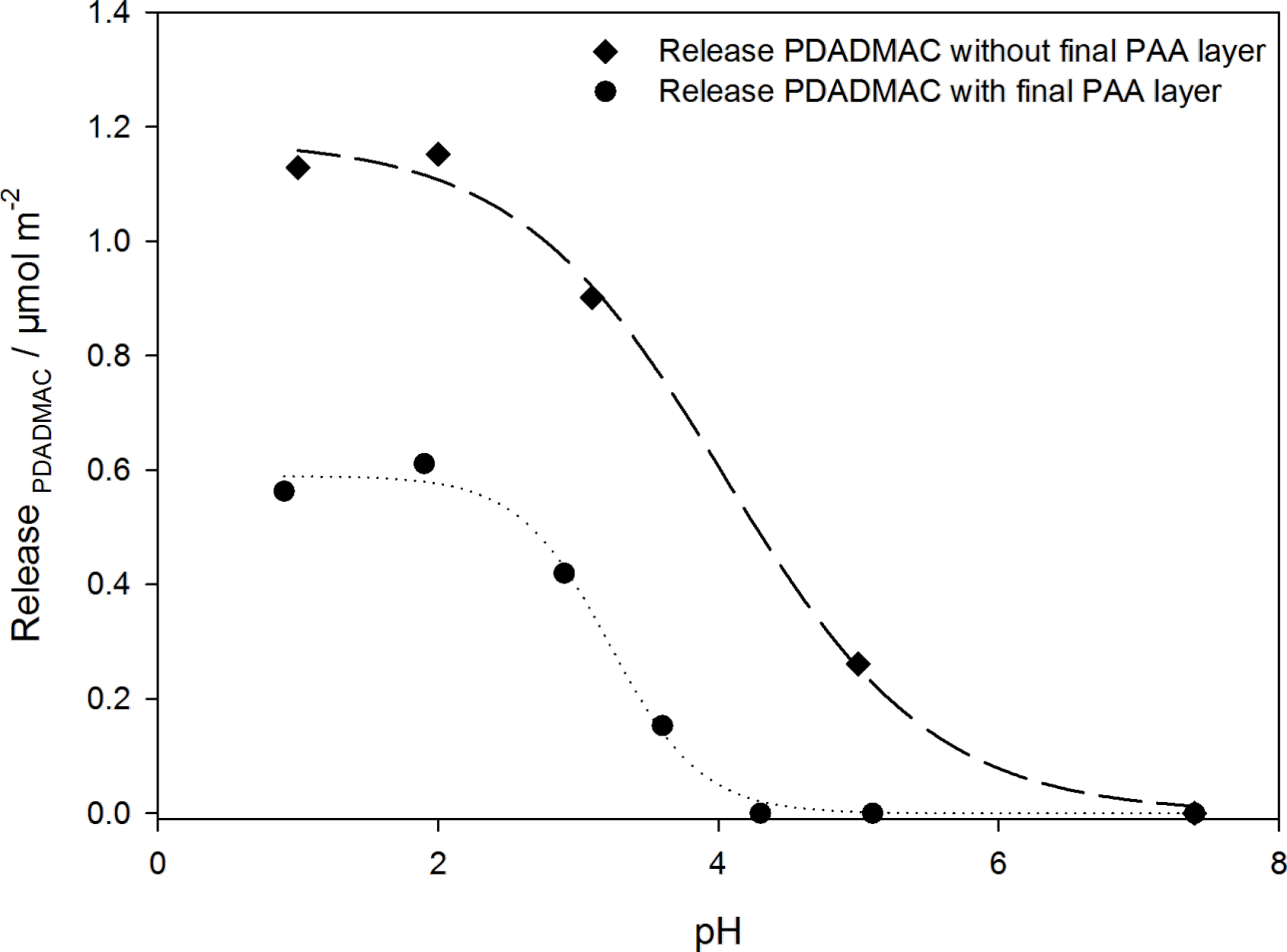
Released amount of PDADMAC per surface area in dependence of the pH. The amount of released PDADMAC was related to the total particle surface to yield the released amount per surface area.

In order to assure the detection of the ^1^H NMR signal of the polymers, the nanoparticles have been prepared using D_2_O as solvent in the two final washing steps. For desorption experiments, the prepared nanoparticle dispersions were adjusted to pH values between pH 7.4 and pH 0.9 before acquisition of ^1^H NMR spectra. As a first step, PLGA-PEI(pH 10)-PAA(pH 5)-PDADMAC(pH 9)-PAA(pH 5) nanoparticles were investigated. Alternatively, PLGA-PEI(pH 10)-PAA(pH 5)-PDADMAC(pH 9) nanoparticles have been prepared, lacking the terminating PAA layer and therefore offering the possibility to investigate the influence of the outer PAA layer on the PDADMAC desorption process. The desorbed amount of PDADMAC in dependence of the adjusted pH is related to the total particle surface and given in Figure 6.

An obvious dependence of the released amount of PDADMAC on the pH can be concluded, as a decrease in pH leads to the release of PDADMAC for both nanoparticle systems investigated. For the nanoparticle system including a terminating PAA layer, the release process starts at pH ≤4 and the released amount reaches a maximum plateau value at about pH 2. The data follow almost exactly a sigmoidal curve and as a release point the pH at 50% release can be determined as 3.2. Interestingly, this release only starts at much lower pH values than expected from previous titration experiments, which show charge neutrality of the PAA layer for the PLGA-PEI(pH 10)-PAA(pH 5) nanoparticle system at pH 4.1, see Figure 2. Since polyelectrolyte layers are dynamic systems of more or less penetrable polyelectrolyte chains, there will always be an amount of charge contributing from underlying layers to the overall electrostatic properties of the polyelectrolyte film. Overall, the total amount of negative charges present at the isoelectric point (IEP) can be concluded to be sufficient in order to preserve a stable PEM, hence preventing PDAMAC to be desorbed from the nanoparticle surface at pH values greater than 4. The release curve for the nanoparticle system lacking the terminating PAA layer also follows a sigmoidal release curve, but PDADMAC desorption already starts at significantly higher pH values compared to the PLGA-PEI(pH 10)-PAA(pH 5)-PDADMAC(pH 9)-PAA(pH 5) nanoparticle system. Additionally, the total amount of released PDADMAC is found to be increased by a factor of almost two. This can be explained considering the degree of ionization of the PAA layer in either nanoparticle system: During build-up of the PLGA-PEI(pH 10)-PAA(pH 5)-PDADMAC(pH 9)-PAA(pH 5) nanoparticles, the pH is reduced from pH 9 after adsorption of PDADMAC to pH 5 during adsorption of the final PAA layer, hence a fraction of PDADMAC may already be released because of the pH decrease during the adsorption of PAA. This also explains that the PDADMAC release is starting at lower pH values in case of the nanoparticle system containing the terminating PAA layer, since the formerly adsorbed amount of PDADMAC is already desorbed during build-up of the terminating PAA layer.

After pH-induced release of the polyelectrolyte we tested for complete release by adding sodium chloride to each sample solution, such that a final concentration of 2 M was reached. Dubas and Schlenoff had previously reported a complete destruction of PDADMAC/PAA multilayers at high salt concentrations (above 0.6 M) [40]. In our systems there was no additional release of PDADMAC observed after the increase of the ionic strength, thus we conclude that a quantitative release can be achieved by pH treatment alone.

## Conclusion

In the present work, we successfully implemented a pH-tunable entity onto biocompatible PLGA nanoparticles using the LbL self-assembly technique and demonstrated ways to control the pH window, in which the nanoparticle surface is pH-sensitive. Successive adsorption of PDADMAC as a cationic compound was accomplished, illustrating the importance to choose adsorption conditions allowing an overcompensation of the nanoparticle surface charge. Furthermore, adsorbed PDADMAC was successfully released after application of an appropriate pH-stimulus, leading to well fitted desorption isotherms. Furthermore, desorption data obtained after omitting the previous adsorption of a terminating PAA layer clearly show a partial desorption of PDADMAC during assembly of the terminating PAA layer, emphasising the pH-sensitivity of PEM consisting of weak polyelectrolytes, such as PAA. The obtained data not only reveal important information about pH-tunability, but also about the absolute stability of polyelectrolyte multilayers and will therefore facilitate future development of applications in the field of pH-sensitive nanostructure assembly.

## Experimental

**Materials:** Poly(lactic-*co*-glycolic acid) (PLGA, Resomer^®^ RG 502H) was purchased from Evonik Industries AG (Darmstadt, Germany). Resorcinol (analytical grade), ethyl acetate (reagent grade; >99.5%), deuterium oxide (D_2_O) (99.9% isotope purity), poly(vinyl alcohol) (PVA) (87–89% hydrolysed; *M*_w_ ≈ 67,000 g/mol), as well as aqueous solutions of polyethylenimine (PEI) (*M*_w_ ≈ 50,000–60,000 g/mol; 50 wt %) and polyacrylic acid (PAA) (*M*_w_ ≈ 100,000 g/mol; 35 wt %) were purchased from Sigma–Aldrich (Steinheim, Germany). An aqueous solution of poly(diallyldimethylammonium chloride) (PDADMAC) (*M*_w_ ≈ 8,500 g/mol; 28 wt %) was purchased from Polysciences (Eppelheim, Germany). Adjustments of pH values were performed by addition of sodium hydroxide solution (1 mol/L) or hydrochloric acid (1 mol/L), which were purchased from Waldeck (Münster, Germany). Washing and dilution steps were carried out using ultrapure water, having a resistivity of at least 18.2 MΩ·cm. All chemicals were used as received without further purification.

**Solutions:** An amount of 500 mg of PVA was dissolved in 50 mL of ultrapure water using gentle heating (60 °C) and magnetic stirring at 500 rpm. The PVA solution was cooled to room temperature and filtered (0.22 µm, cellulose acetate filter unit) directly before use. Additionally, 500 mg of PLGA were dissolved in 5 mL ethyl acetate. Polyelectrolyte solutions were diluted to concentrations of 10 mmol/L (PAA and PDADMAC) and 20 mmol/L (PEI), respectively. All polyelectrolyte concentrations were calculated with respect to the monomeric unit of the corresponding compound. If needed, pH was adjusted by addition of 1 molar hydrochloric acid (HCl) and 1 molar sodium hydroxide solution (NaOH).

**Dynamic light scattering (DLS):** Average particle size, polydispersity, and zeta potential were determined using a Zetasizer Nano ZS (Malvern Instruments, UK). All measurements were performed at ambient temperature of (22 ± 0.1) °C in ultrapure water as dispersant, unless mentioned otherwise. For titration experiments, a multi-purpose titration device MPT-2 (Malvern Instruments, UK) was connected to the Zetasizer Nano ZS. Titrations were carried out by diluting 5 mg nanoparticles (calculated as dry solids content) with ultrapure water to a final volume of 12 mL. Hydrochloric acid (25 mM) was chosen as the corresponding titration agent. During titration experiments, the pH was decreased step-wise to a final value of 2.5, while particle diameter, polydispersity, and zeta potential were monitored for each pH step.

**Gravimetric analysis:** Determination of nanoparticle concentration in dispersion was carried out by gravimetric analysis using a Sartorius SE2 Ultra microbalance (Sartorius AG, Göttingen, Germany). An aliquot (20.0 µL) of homogenized nanoparticle dispersion was transferred to a disposable aluminium weighing dish, which was weighted previously. The filled weighing dish was dried for at least four hours in a drying cabinet (Thermo Fisher Scientific Inc., USA) at 80 °C. The particle concentration of the initial dispersion was derived from the weight of the solids content.

**Nuclear magnetic resonance (NMR) spectroscopy:** ^1^H NMR experiments were performed using a 400 MHz Avance spectrometer (Bruker, Rheinstetten, Germany). All measurements were carried out at room temperature (295 K) and all sample solutions were prepared in D_2_O. For quantitative NMR measurements of PDADMAC, a solution of resorcinol in D_2_O was used as an external standard. It was filled into a melting point capillary, which was flame-sealed on both ends and inserted on the central axis of an NMR tube containing the sample solution. A calibration was carried out by relating the resorcinol signal at 7.1 ppm to the PDADMAC signals (at 3.15 and 3.25 ppm) of solutions with known PDADMAC concentrations (1 mM and 2 mM). This obtained calibration was used to determine the amount of PDADMAC desorbed from nanoparticles after applying a pH stimulus.

**Nanoparticle preparation:** PLGA nanoparticles were prepared by a modified emulsion diffusion method. The addition of a stabilizing agent is generally favoured due to increased final particle stability. Here, poly(vinyl alcohol) (PVA) was used for particle preparation since it has proven to have outstanding emulsification properties and a low toxicity at the same time. In brief, 500 mg of PLGA was dissolved in 5 mL ethyl acetate. After addition of 10 mL aqueous PVA solution (10 mg/mL), the obtained emulsion was mixed using an Ultra Turrax T25 homogenization device (IKA Werke GmbH & Co KG, Staufen, Germany) at 17,000 rpm for 5 min to ensure a small and homogeneous droplet formation. The obtained emulsion was added to 40 mL of PVA solution (10 mg/mL) and stirred at 550 rpm at room temperature for at least 12 h in order to completely evaporate the ethyl acetate phase, leading to final nanoparticle precipitation. After particle formation, the dispersion was washed by centrifugation (Centrifuge 5242, Eppendorf, Germany) and removal of the supernatant containing excess PVA. The procedure was followed by redispersion in ultrapure water, treatment by a vortexing device (VWR VV3, Darmstadt, Germany) and gentle sonication (Bandelin Sonorex PK 102 H, Berlin, Germany), if necessary. The washing step was performed three times in order to completely remove the PVA from the aqueous phase.

**Layer-by-layer (LbL) self-assembly:** Polyelectrolyte multilayers were adsorbed to PLGA nanoparticles using the LbL self-assembly technique, as described elsewhere [15,41–42]. For each adsorption step, polymer solution was stirred at 700 rpm using a magnetic stirrer, while an equal volume of nanoparticle dispersion (10 mg/mL) was added dropwise. To ensure complete polymer adsorption, an incubation time of 20 min was chosen. Excess polymer was washed out by centrifugation at 4,000*g* for 20 min and removal of the supernatant. The particles were then redispersed in ultrapure water, using sonication and a vortexer, if necessary. By this procedure, particle dispersions were washed twice after each adsorption step.
